# Engineering central pathways for industrial-level (3*R*)-acetoin biosynthesis in *Corynebacterium glutamicum*

**DOI:** 10.1186/s12934-020-01363-8

**Published:** 2020-05-12

**Authors:** Lingxue Lu, Yufeng Mao, Mengyun Kou, Zhenzhen Cui, Biao Jin, Zhishuai Chang, Zhiwen Wang, Hongwu Ma, Tao Chen

**Affiliations:** 1 Frontier Science Center for Synthetic Biology and Key Laboratory of Systems Bioengineering of Ministry of Education, SynBio Research Platform, Collaborative Innovation Center of Chemical Science and Engineering, School of Chemical Engineering and Technology, Tianjin University, Tianjin, 300072 China; 2 Key Laboratory of Systems Microbial Biotechnology, Tianjin Institute of Industrial Biotechnology, Chinese Academy of Sciences, Tianjin, 300308 China

**Keywords:** (3*R*)-Acetoin, *Corynebacterium glutamicum*, Metabolic engineering, Microbial fermentation, Citrate synthase, Green chemistry

## Abstract

**Background:**

Acetoin, especially the optically pure (3*S*)- or (3*R*)-enantiomer, is a high-value-added bio-based platform chemical and important potential pharmaceutical intermediate. Over the past decades, intense efforts have been devoted to the production of acetoin through green biotechniques. However, efficient and economical methods for the production of optically pure acetoin enantiomers are rarely reported. Previously, we systematically engineered the GRAS microorganism *Corynebacterium glutamicum* to efficiently produce (3*R*)-acetoin from glucose. Nevertheless, its yield and average productivity were still unsatisfactory for industrial bioprocesses.

**Results:**

In this study, cellular carbon fluxes in the acetoin producer CGR6 were further redirected toward acetoin synthesis using several metabolic engineering strategies, including blocking anaplerotic pathways, attenuating key genes of the TCA cycle and integrating additional copies of the *alsSD* operon into the genome. Among them, the combination of attenuation of citrate synthase and inactivation of phosphoenolpyruvate carboxylase showed a significant synergistic effect on acetoin production. Finally, the optimal engineered strain CGS11 produced a titer of 102.45 g/L acetoin with a yield of 0.419 g/g glucose at a rate of 1.86 g/L/h in a 5 L fermenter. The optical purity of the resulting (3*R*)-acetoin surpassed 95%.

**Conclusion:**

To the best of our knowledge, this is the highest titer of highly enantiomerically enriched (3*R*)-acetoin, together with a competitive product yield and productivity, achieved in a simple, green processes without expensive additives or substrates. This process therefore opens the possibility to achieve easy, efficient, economical and environmentally-friendly production of (3*R*)-acetoin via microbial fermentation in the near future.

## Background

Acetoin (3-hydroxy-2-butanone) is a popular food additive with a pleasant butter-like flavor [1]. Since it can be obtained from biomass and possesses reactive carbonyl and hydroxyl moieties, the United States Department of Energy designated acetoin as one of 30 promising platform chemicals that were given priority for development and utilization in 2004 [2]. Acetoin is chiral, and its enantiomers (3*S*)- and (3*R*)-acetoin are more valuable than the racemate as they are potential pharmaceutical intermediates [3]. Moreover, they can be used to synthesize liquid crystal materials and novel optically active α-hydroxyketone derivatives [4].

At present, the commercial production of acetoin is mostly based on chemical methods with many disadvantages, such as high cost, high pollution, and low yield [5]. Moreover, the use of chemosynthetic, non-natural acetoin in food and cosmetics is restricted due to safety concerns [6, 7]. The biotechnological production of safe and natural acetoin could be more ecological and sustainable than their chemical counterparts [8, 9]. These methods, including microbial fermentation [8, 10, 11], whole-cell biocatalysis [12, 13] and enzymatic biocatalysis [14, 15], have consequently gained great attention over the past decades. *Saccharomyces cerevisiae* JHY617-SDN was able to efficiently accumulate 100.2 g/L acetoin from glucose during fed-batch fermentation [16]. *Gluconobacter oxydans* NL71 could produce 165.9 g/L acetoin from 2,3-butanediol in a whole-cell catalysis process [17]. This is the highest acetoin titer ever reported. However, the enantiomeric excess of the produced acetoin was not reported, and efficient methods for the production of optically pure (3*R*)-acetoin were rarely reported. Nevertheless, some representative publications with good results do exist. Using a two-enzyme coupling system, 12.2 g/L of optically pure (3*S*)-acetoin was efficiently produced from diacetyl with a high productivity of 9.76 g/L/h [18]. By constructing an efficient *E. coli* whole-cell biocatalyst, Guo et al. [19] obtained a high (3*R*)-acetoin titer of 86.7 g/L from optically pure (*R*,*R*)-2,3-butanediol. However, the expensive chiral substrate, and the complicated, costly processing steps, such as protein purification through Ni–NTA affinity chromatography or centrifugation to concentrate the catalyst cells, made it economically unfeasible in industrial applications.

Acetoin production from petrochemicals such as the expensive chiral 2,3-bantediol or the noxious diacetyl is costly and unsustainable. Consequently, (3*R*)-acetoin production from renewable substrates via microbial fermentation is highly favored in recent studies. Many microorganisms can naturally synthesize optically pure (3*R*)-acetoin, including *Klebsiella pneumoniae* [20], *Serratia marcescens* [21, 22], *Lactococcus lactis* [23] and *Bacillus* sp. [24]. When the acetoin degradation pathway was disrupted, the *Klebsiella pneumoniae* strain Δ*budC*Δ*aco* was able to accumulate 62.3 g/L (3*R*)-acetoin in 57 h [20]. However, this pathogenic microorganism can be hazardous to humans, which entails unacceptable safety risks in industrial-scale production. Dai et al. [25] isolated the marine strain *Bacillus subtilis* CGMCC 13141 capable of producing 83.7 g/L of (3*R*)-acetoin with a high yield of 0.447 g/g glucose in fed-batch fermentation. However, the titer was still insufficient for industrial demands.

**Fig. 1:**
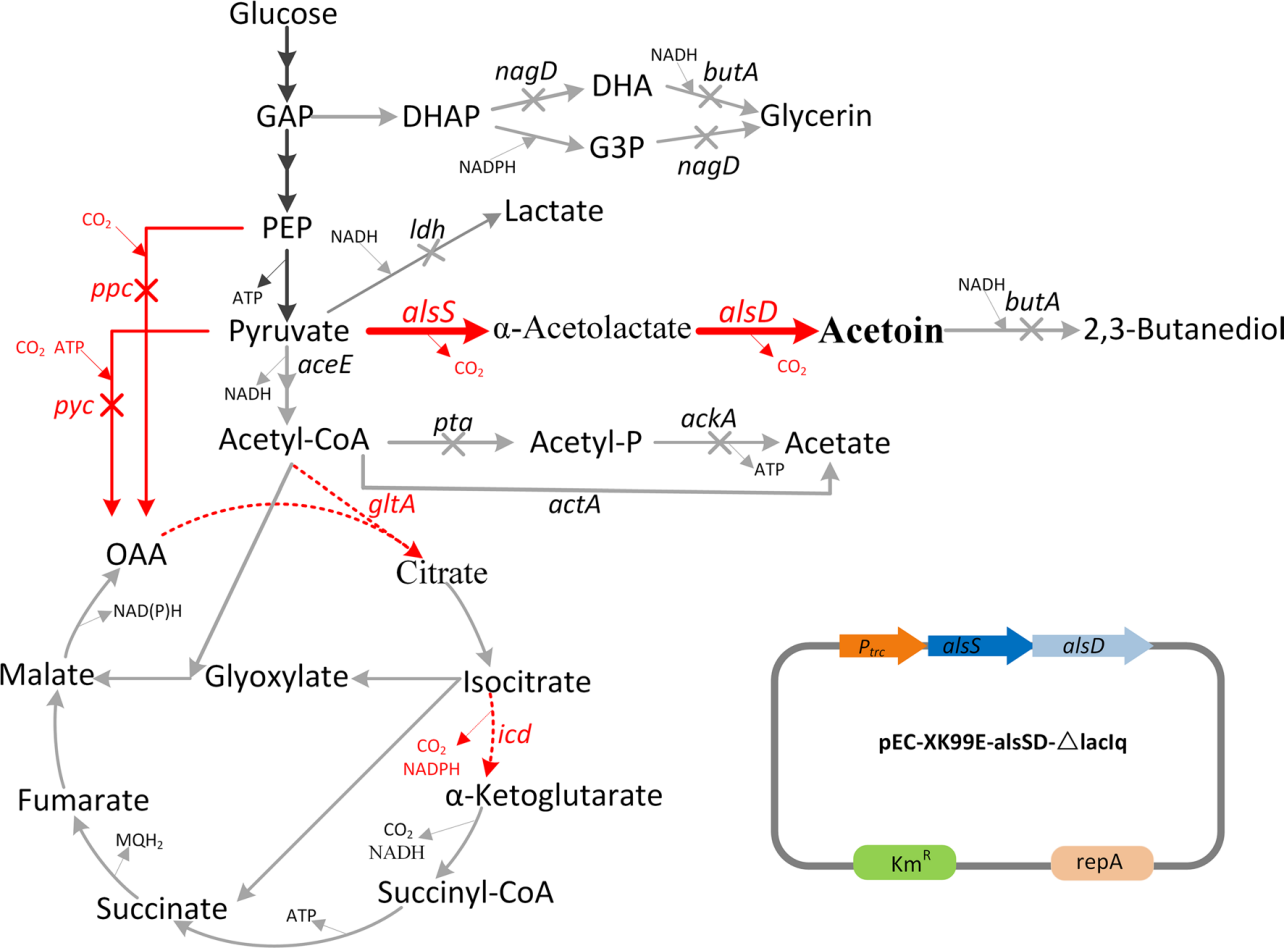
The (3*R*)-acetoin biosynthesis pathway of *C. glutamicum.* Genes manipulated in this study are indicated in red. The bold arrows indicate metabolic fluxes increased by overexpression of the corresponding genes. The gray arrows indicate the reactions leading to a byproduct or presumably irrelevant reactions. Deleted genes are indicated with crosses. Downregulated genes are indicated with dashed arrows. Abbreviations: GAP, glyceraldehyde-3-phosphate; DHAP, dihydroxyacetone phosphate; DHA, dihydroxyacetone; G3P, *sn*-glycerol 3-phosphate; PEP, phosphoenolpyruvate; OAA, oxaloacetate. Genes and their encoded enzymes: *alsS,* acetolactate synthase; *alsD,* acetolactate decarboxylase*; ppc,* phosphoenolpyruvate carboxylase; *pyc,* pyruvate carboxylase; *icd,* isocitrate dehydrogenase; *gltA*, citrate synthase. *pta*, phosphotransacetylase; *ackA*, acetate kinase; *aceE*, E1 component of the pyruvate dehydrogenase complex; *nagD*, putative phosphatase; *butA*, 2,3-butanediol dehydrogenase

In our previous study [26], we systematically engineered *Corynebacterium glutamicum*, the famous amino acids industrial workhorse with GRAS (generally recognized as safe) status, to develop a safe and efficient industrial (3*R*)-acetoin producer. The best strain CGR7 was able to produce 96.2 g/L (3*R*)-acetoin with an optical purity of more than 95% in fed-batch mode with a yield of 0.360 g/g glucose and productivity of 1.30 g/L/h [9], highlighting *C. glutamicum* as a competitive producer of enantiopure acetoin with fantastic potential for industrial-level production. While this was still the highest reported titer at the time of writing, the corresponding yield (73.5% of the theoretical value) and average productivity were still unsatisfactory for economical production. In this study, the previously engineered strain CGR6 (ATCC13032 ∆*pta*∆*ackA*∆*ldh*∆*butA*∆*nagD*; ∆*ackA*::P_*tuf*_ -*alsSD*) [9] was chosen as a chassis for further optimization. In this strain, the biosynthesis pathways of the major by-products lactate, glycerin, and acetate, as well as the downstream product 2,3-butanediol were disrupted to increase the pool of the precursor pyruvate and prevent acetoin from being reduced to downstream 2,3-butanediol. Moreover, a copy of the acetoin synthesis operon *alsS*-*alsD* under the control of the strong constitutive promoter *P*_*tuf*_, was inserted into the locus of the deleted gene *ackA* to enhance acetoin production. Here, several strategies were successively applied to redirect more carbon flux toward acetoin synthesis in strain CGR6, including interdicting anaplerotic pathways, weakening key genes of the TCA cycle, and inserting additional copies of the *alsSD* operon into the genome of the host. The optimal engineered strain CGS11 (Fig. 1) achieved a titer of 102.45 g/L acetoin with a yield of 0.419 g/g glucose at a rate of 1.86 g/L/h in fed-batch fermentation.

## Materials and methods

### Reagents, strains and media

Primers were synthesized by GENEWIZ (Suzhou, China). Plasmids were extracted using the Axyprep™ Plasmid Miniprep Kit (Axygen, USA) and the SanPrep Column Plasmid Mini-Prep Kit (Sangon Biotech, Shanghai, China). BHI Broth was purchased from Hopebio (Qingdao, China). Yeast extract was purchased from Angel (Hubei, China). Acetoin, creatine, 1-naphthol, ethyl 2-acetoxy-2-methylacetoacetate, oxaloacetate and 5,5′-dithiobis(2-nitrobenzoic) acid were purchased from Sigma (Merck, USA). The 2,3,5,6-tetramethylpyrazine standard was purchased from Tokyo Chemical Industry (Tokyo, Japan). Other reagents were purchased from Sangon Biotech (Shanghai, China).

The strains and plasmids used in this study were listed in Table 1. *Escherichia coli* DH5α was used for plasmid construction and was grown in lysogeny broth (LB) medium containing (per liter) 10 g tryptone, 5 g yeast extract, and 10 g NaCl. Brain heart infusion (BHI) medium was used for the transformation and tube culture of *C. glutamicum*. CGIII medium composed of (per liter) 10 g tryptone, 10 g yeast extract, 2.5 g NaCl and 20 g glucose was used for pre-cultures of *C. glutamicum*. Batch fermentation of acetoin was conducted in CGXIIP medium containing (per liter): 10 g yeast extract, 5 g (NH_4_)_2_SO_4_, 5 g urea, 1 g KH_2_PO_4_, 1 g K_2_HPO_4_, 0.25 g MgSO_4_·7H_2_O, 0.01 g CaCl_2_, 0.01 g FeSO_4_·7H_2_O, 0.1 mg MnSO_4_·H_2_O, 1 mg ZnSO_4_·7H_2_O, 0.2 mg CuSO_4_·5H_2_O, 0.02 mg NiCl·6H_2_O, 0.4 mg biotin and 38 g glucose, pH 7.0. Fed-batch fermentation was conducted in LBRC medium containing (per liter): 10 g yeast extract, 50 g corn steep liquor, 1 g urea, 0.5 g K_2_HPO_4_, 0.5 g MgSO_4_·7H_2_O and 2 g sodium acetate, supplemented with the indicated amount of glucose, pH 7.0. The stock comprising 1000 g/L glucose used for fed-batch fermentation, was prepared by putting 100 g glucose and 36 mL ddH_2_O in a Schott-Duran bottle and sterilizing at 110 °C for 10 min, then stored at 60 °C. Antibiotics were added where appropriate as follows: for *C. glutamicum*, kanamycin 25 mg/L, for *E. coli*, kanamycin 40 mg/L.

**Table 1:** Strains and plasmids used in this study

Strain/plasmid	Relevant characteristics	References
*E. coli DH5α*	Host for plasmid cloning	Invitrogen
ATCC 13032	*C. glutamicum* wild type Biotin auxotrophic	ATCC^a^
CGR6	ATCC13032*∆pta∆ackA∆ldh∆butA∆nagD, ∆ackA::P*_*tuf*_ -*alsSD*	[9]
CGS1	CGR6 *∆ppc*	This study
CGS2	CGR6 *∆pyc*	This study
CGS3	CGR6 *∆ppc∆pyc*	This study
CGS4	CGS3 ICD^A94D^	This study
CGS5	CGS3 ICD^G407S^	This study
CGS6	CGS3 ICD^R453*S*^	This study
CGS7	CGR6 *P*_*1*_-*gltA*	This study
CGS8	CGS1 *P*_*1*_-*gltA*	This study
CGS9	CGS8 *∆butA::P*_*tuf*_ -*alsSD, ∆nagD::P*_*tuf*_ -*alsSD*	This study
CGS11	CGS9 pEC-XK99E-*alsSD*-*∆lacIq*	This study
Plasmids		
pD-*sacB*	Kan^R^; vector for in-frame deletion (*sacB*_*B.sub*_.; *lacZα*; *OriV*_*E.c*_.)	[56]
pD-*sacB*-*ppc*	pD-*sacB* carrying the flanking sequences of the *ppc* gene	This study
pD-*sacB*-*pyc*	pD-*sacB* carrying the flanking sequences of the *pyc* gene	This study
pD-*sacB*-*butA*	pD-*sacB* carrying the flanking sequences of the *butA* gene	[41]
pD-*sacB*-*nagD*	pD-*sacB* carrying the flanking sequences of the *nagD* gene	[9]
pD-*sacB*-*icd*-mut-1	Kan^R^, containing the nucleotide sequence for ICD amino acid exchange A94D	This study
pD-*sacB*-*icd*-mut-2	Kan^R^, containing the nucleotide sequence for ICD amino acid exchange G407S	This study
pD-*sacB*-*icd*-mut-3	Kan^R^, containing the nucleotide sequence for ICD amino acid exchange R453S	This study
pD-*sacB*-*P*_*1*_-*gltA*	Kan^R^, containing P_1_ promoter and *gltA* flanks	Unpublished work
pD-*sacB*-*butA*-*alsSD*	Kan^R^, containing *P*_*tuf*_ -*alsSD* flanks	This study
pD-*sacB*-*nagD*-*alsSD*	Kan^R^, containing *P*_*tuf*_ -*alsSD* flanks	This study
pEC-XK99E	Kan^R^; *C. glutamicum*/*E. coli* shuttle vector (*P*_*trc*_, *lacIq*; pGA1, *OriV*_*C.g.*_, *OriV*_*E.c.)*_	[57]
pEC-XK99E-*alsSD*-Δ*lacIq*	Derived from pEC-XK99E, for the overexpression of *alsS* and *alsD* under promoter *P*_*trc*_	[9]

^a^ ATCC, American Type Culture Collection

### Construction of plasmids and strains

All the primers used in this study are listed in supplementary Additional file 1: Table S1. All DNA manipulations, including restriction enzyme digestion and vector isolation were carried out using standard protocols [27]. The suicide plasmid pD-*sacB* was used for genome editing in *C. glutamicum* via two-step homologous recombination [28].

To delete the *pyc* gene, the vector pD-*sacB*-*pyc* was constructed as follows: the upstream sequence of *pyc* and a *pyc*-specific fragment were amplified from *C. glutamicum* genomic DNA by PCR using the primer pairs pyc-1/pyc-2 and pyc-3/pyc-4, respectively, and fused by PCR. The resulting product was digested with *BamH*I and *Sal*I and ligated between the corresponding sites of pD-*sacB*. The plasmid pD-*sacB*-*ppc* was constructed analogously, using *BamH*I and *Hind*III.

To introduce mutations into the endogenous gene *icd*, the plasmid pD-*sacB*-*icd*-mut-1 was constructed as follows: the flanking regions of the *icd* gene with relevant modifications were amplified from genomic DNA of *C. glutamicum* using the primer pairs ICD^A94D^-1/ICD^A94D^-2 and ICD^A94D^-3/ICD^A94D^-4. The corresponding flanking fragments were fused using ICD^A94D^-1/ICD^A94D^-4. The fused product was digested with *BamH*I and *Sal*I and ligated between the corresponding sites of pD-*sacB* to construct pD-*sacB*-*icd*-mut-1. The plasmids pD-*sacB*-*icd*-mut-2 and pD-*sacB*-*icd*-mut-3 were constructed analogously, using *Sma*I and *Sal*I.

To integrate additional copies of the acetoin operon into the chromosome, the plasmid pD-*sacB*-*butA*-*alsSD* was constructed as follows: the acetoin operon (*alsS*-*alsD*) was amplified from the genome of CGR6 using the primer pair BalsSD-1/BalsSD-2. The resulting fragment was digested with *Xho*I/*Sal*I and ligated between the corresponding sites of pD-*sacB*-*butA*. The plasmid pD-*sacB*-*nagD*-*alsSD* was constructed analogously.

### Fermentation conditions

To prepare *C. glutamicum* pre-cultures, single colonies were used to inoculate 5 mL of BHI medium and grown at 30 °C and 220 rpm overnight, after which the entire resulting culture was used to inoculate 50 mL of CGIII medium, and grown to an OD_600_ of 10. For batch fermentation, the seed culture was used to inoculate a 250-mL shake flask containing 50 mL of CGXIIP medium to an initial OD_600_ of 1 and grown at 30 °C and 220 rpm on a rotary shaker.

For fed-batch fermentation, 100 mL of LBRC seed culture was used to inoculate a 5-L fermenter (Bailun, Shanghai, China) containing 1.8 L of LBRC medium. All cultivations were carried out at 30 °C with an aeration rate of 1 vvm. The agitation speed was maintained at 600 rpm. The initial pH of the medium was 6.5. During the fermentation process, the pH value was not controlled. The initial glucose concentration was 50 g/L, and an appropriate amount of 1000 g/L glucose stock was added to maintain its concentration between 10 and 50 g/L.

### Analytical methods

The biomass concentration was calculated from OD_600_ values using an experimentally determined correlation 1 OD_600_ unit is equal to 0.25 g/L cell dry weight (CDW) [29]. Glucose was measured using an SBA Bio-analyzer (Shandong Academy of Sciences, China) after appropriate dilution. Metabolite concentrations were determined by HPLC, using an HPX-87H (300 mm × 7.8 mm) organic acid and sugar analysis column (Bio-Rad, China), kept at 60 °C, and a refractive index detector. The injection volume was 10 μL, and the mobile phase consisted of 5 mM H_2_SO_4_ at a flow rate of 0.4 mL/min. The concentration of 2,3,5,6-tetramethylpyrazine was analyzed by GC-FID (PERSEE, Beijing, China) equipped with an HP-5 (19091 J-413, 30 m × 0.32 mm) capillary column (Agilent, USA) as described previously [30]. The optical purity of acetoin was determined by GC as described previously [9]. The fermentation broth was quenched by 40% pre-cooled (− 40 °C) methanol and centrifuged at 5000 × g for 1 min immediately to harvest cells, then resuspended cells with extracting solution provided by the Pyruvate Assay Kit (Solarbio, Beijing, China) and disrupted cells by ultrasonication. The reaction, including the construction of pyruvate standard curves, was carried out using the Pyruvate Assay Kit.

### Enzyme activity assays

Cells of the engineered strains were grown to the middle exponential phase (12 h) and late exponential phase (22 h), after which the fermentation broth was centrifuged at 5000×*g* for 10 min to harvest cells, which were washed twice with 1 mL 200 mM phosphate buffered saline (pH 7.0) and then resuspended in 0.3 mL of the same buffer. The cells were disrupted by ultrasonication and cell debris was removed by centrifugation at 5000×*g* and 4 °C for 10 min. Enzyme activity was assayed in the resulting supernatant. Total protein concentrations were determined according to the Bradford method [31]. The formed amount of acetoin generated by acetolactate was measured to determine the acetolactate synthase (ALS) activity as described previously [32]. The acetolactate decarboxylase (ALDC) activity was assayed by measuring the production of acetoin as described previously [33]. The citrate synthase activity was determined by measuring the amount of CoA formed as described previously [34].

## Results and discussion

### Improvement of acetoin production by deleting *ppc*/*pyc* to reduce succinate accumulation

**Fig. 2:**
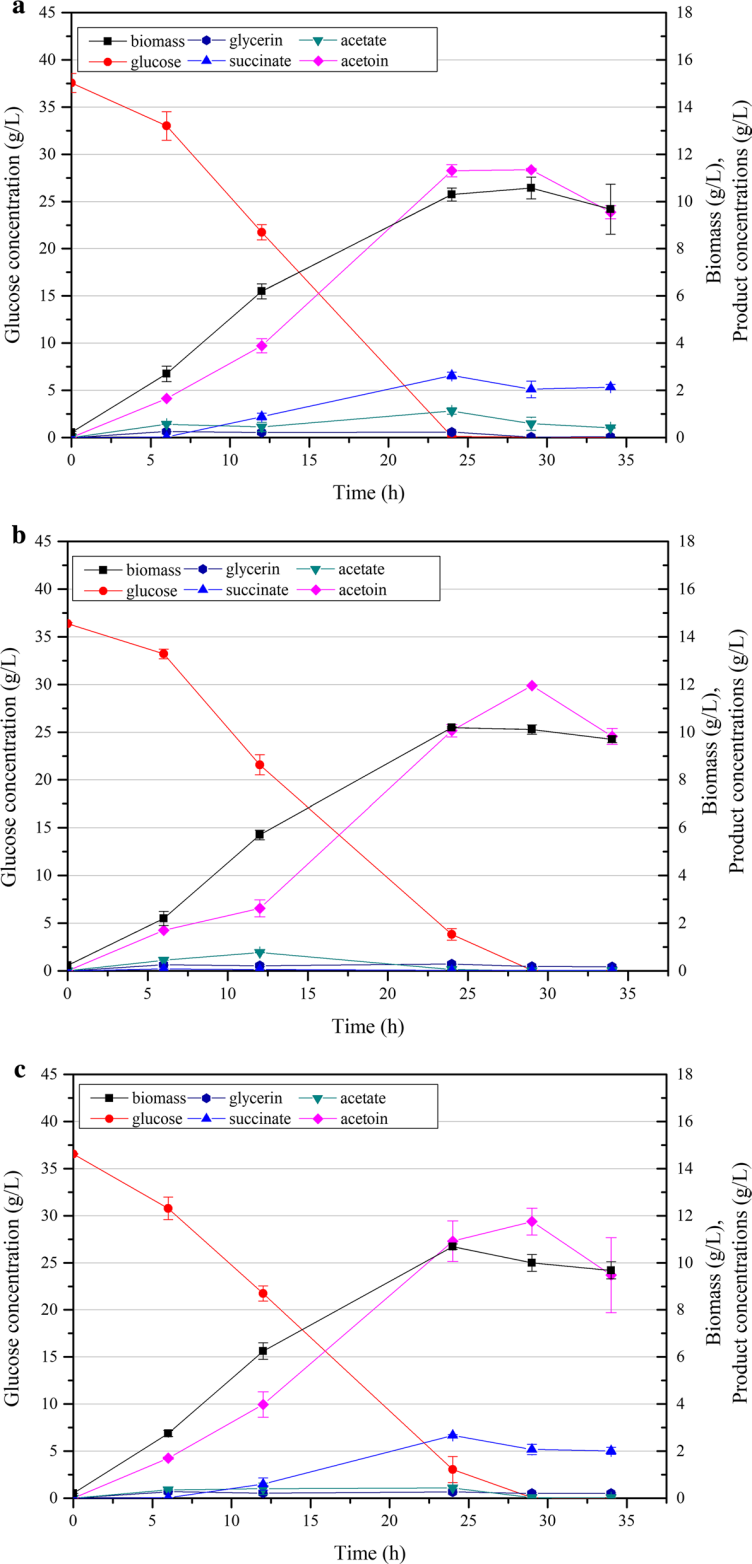
Time profiles of the biomass (g/L), glucose, organic acid and acetoin concentrations of strains CGR6 (**a**), CGS1 (**b**) and CGS2 (**c**) cultured with 38 g/L glucose. Error bars indicate the standard deviations from three independent cultures

To analyze the carbon distribution in strain CGR6, batch fermentation in flasks was carried out. As shown in Fig. 2a, CGR6 was able to produce 11.30 g/L acetoin when glucose was almost depleted at 24 h, corresponding to a yield of 0.302 g/g glucose. As expected, no 2,3-butandiol or lactate was detected, since their synthesis pathways were blocked. Although 0.52 g/L α-ketoglutarate was detected when fermenting this strain in CGXIIY medium [9], it was not detected in CGXIIP medium in this study. The major by-product was succinate, with a titer of 2.63 g/L, followed by acetate (1.12 g/L) and glycerin (0.23 g/L). In our previous work, two key genes of succinate synthesis, *ppc* encoding phosphoenolpyruvate (PEP) carboxylase and *pyc* encoding pyruvate carboxylase, were respectively deleted via one-step homologous recombination (single crossover) with a tetracycline resistance marker [9]. The *ppc*/*pyc*-deficient strain showed an improved acetoin production in batch fermentation in flasks. However, the use of the tetracycline resistance marker impeded further genetic manipulation and was not acceptable for an industrial producer. In this study, we deleted the *ppc* and *pyc* genes in CGR6 using a markerless two-step recombination method (double crossover) [35], resulting in the strains CGS1 and CGS2, respectively.

As shown in Fig. 2b, succinate production was almost completely abolished in the *ppc* knockout strain CGS1, with a titer of only 0.03 g/L. The glucose consumption rate of CGS1 was decreased compared with that of CGR6, and 11.96 g/L of acetoin was obtained when glucose was exhausted at 29 h. This corresponds to a yield of 0.328 g/g glucose, which was 9.3% higher than that of CGR6. By contrast, the succinate production in the *pyc* knockout strain CGS2 was almost unchanged (Fig. 2c), but its acetoin production was enhanced with a titer of 11.75 g/L when glucose was depleted at 29 h, and a yield of 0.323 g/g glucose. The glucose consumption rate of CGS2 was also decreased compared with that of CGR6, but it was somewhat higher than that of CGS1 at 24 h.

Mutants deficient in *ppc* rather than *pyc* could efficiently reduce succinate accumulation, indicating that PEP rather than pyruvate is the key precursor of succinate under aerobic conditions. In agreement with previous reports [36, 37], the growth of CGS1 and CGS2 was only mildly decreased, but their acetate titers (respectively 0.06 and 0.44 g/L) were unexpectedly significantly decreased compared with that of CGR6 (1.12 g/L) (Table 2). We suspected that the reduced acetate accumulation in CGS1 and CGS2 was resulted from the decreased glucose consumption rates (Table 2), which can reduce the flux in overflow metabolism. The only another detected by-product was glycerin, with a titer of about 0.30 g/L in both CGS1 and CGS2. The two strains both showed improved acetoin yields, which was consistent with our previous results [9]. However, strain CGS3 with *ppc* and *pyc*-deficient was unable to grow on glucose in mineral medium and still grew poorly with 10 g/L yeast extract addition (data not shown), which was consistent with previous reports [37, 38]. After considering the acetoin titer, yield and by-products accumulation, strain CGS1 was chosen for further manipulation.

**Table 2:** Fermentation characteristics of *C. glutamicum* strains cultivated in CGXIIP medium supplemented with initial 38 g/L glucose measured at 24 h

Strain	Biomass (g/L)	Consumed glucose (g/L)	Acetoin yield (g/g glucose)	Acetoin productivity (g/L/h)	Titer (g/L)			
					Acetoin	Acetate	Glycerin	Succinate
CGR6	10.29 ± 0.27	37.42 ± 0.06	0.302 ± 0.009	0.47 ± 0.01	11.30 ± 0.26	1.12 ± 0.14	0.23 ± 0.02	2.63 ± 0.15
CGS1	10.19 ± 0.03	32.54 ± 0.59	0.309 ± 0.005	0.42 ± 0.01	10.06 ± 0.26	0.06 ± 0.01	0.29 ± 0.01	0.03 ± 0.01
CGS2	10.18 ± 0.11	33.50 ± 1.39	0.326 ± 0.020	0.45 ± 0.04	10.91 ± 0.86	0.44 ± 0.04	0.27 ± 0.03	2.67 ± 0.22
CGS5	8.67 ± 0.34	24.55 ± 1.08	0.272 ± 0.009	0.28 ± 0.02	6.68 ± 0.54	0.72 ± 0.07	0.32 ± 0.01	<0.01
CGS7	9.58 ± 0.21	35.84 ± 1.43	0.337 ± 0.011	0.50 ± 0.01	12.07 ± 0.38	0.58 ± 0.03	0.20 ± 0.03	2.02 ± 0.13
CGS8	9.34 ± 0.12	30.17 ± 0.98	0.415 ± 0.005	0.52 ± 0.01	12.53 ± 0.03	0.15 ± 0.01	0.17 ± 0.03	0.03 ± 0.02
CGS9	7.88 ± 0.45	24.45 ± 1.52	0.484 ± 0.015	0.49 ± 0.01	11.83 ± 0.35	0.29 ± 0.03	0.08 ± 0.01	0.03 ± 0.02
CGS11	8.06 ± 0.44	24.54 ± 0.46	0.497 ± 0.005	0.51 ± 0.01	12.22 ± 0.20	0.14 ± 0.01	0.05 ± 0.01	0.03 ± 0.01

Error bars indicate the standard deviations from three independent cultures

### Improvement of acetoin production by blocking anaplerotic pathways and introducing isocitrate dehydrogenase mutants

Most studies on improving precursor pyruvate (Fig. 1) availability focused on complete inactivation or attenuation of the pyruvate dehydrogenase complex (PDHC) by deleting the *aceE* gene or reducing its promoter activity [39, 40]. In our previous work, the *aceE* gene was also deleted to conserve pyruvate and improve acetoin production. However, additional acetate was required for cell growth and the best strain CGL3 (*C. glutamicum* ∆*aceE*∆*ldh*∆*butA*; pEC-XK99E-*alsSD*) could only accumulate 8.33 g/L acetoin from about 33 g/L glucose and 10 g/L acetate under optimal conditions [41]. In cases of attenuating PDHC, although the growth of the resulting strain was independent of acetate addition, the cell growth and glucose consumption rate were dramatically reduced [39]. Therefore, deletion or attenuation of PDHC might not be optimal for manufacturing optically pure (3*R*)-acetoin.

A double deletion of *ppc* and *pyc* is generally lethal for *C. glutamicum* due to a lack of oxaloacetate [37]. However, a novel strategy for improving the biosynthesis of pyruvate-derived metabolites by introducing newly identified isocitrate dehydrogenase mutants (A94D, G407S or R453*S*) was recently proposed [38]. These *icd* mutations both lowered the ICD activity and activated the glyoxylate shunt, and were consequently able to recover oxaloacetate re-supply and cell growth of *C. glutamicum* ∆*ppc*∆*pyc* [38]. Thus, three ICD mutants A94D, G407S and R453S were introduced into CGS3 to test their effects on acetoin production, resulting in strains CGS4, CGS5 and CGS6, respectively. As shown in Additional file 1: Fig. S1, strains CGS4 and CGS6 showed almost the same growth inhibition as strain CGS3. This is probably because strain CGS3 is an acetoin producing host, in which the major carbon fluxes have already been redirected toward acetoin synthesis, and are therefore insufficient to support an activated glyoxylate shunt to re-supply oxaloacetate. Nevertheless, CGS5 with the mutant ICD^G407S^ successfully recovered cell growth to some extent. However, acetoin production in CGS5 was dramatically decreased to a titer of only 11.37 g/L when glucose was exhausted at 34 h, and a yield of 0.280 g/g glucose, which was 14.6% lower than that of CGS1 (Additional file 1: Fig. S2). This indicated that the carbon fluxes from acetoin synthesis rather than its competing pathways were redirected to regain biomass synthesis, but the reason for this is still unknown.

### Improvement of acetoin production by reducing citrate synthase activity

Since directly blocking acetyl-CoA or oxaloacetate synthesis severely affected the growth performance and failed to improve acetoin production, the focus of engineering was moved to citrate synthase (CS, encode by *gltA*), which condenses acetyl-CoA and oxaloacetate to citrate and is one of the most important sinks for the flux of these two precursors. Therefore, reduction of CS activity was viewed as a promising strategy to lower the synthesis of acetyl-CoA and oxaloacetate, and thereby conserve pyruvate for improved acetoin production. *C. glutamicum* mutants devoid of CS were unable to grow on glucose [34]. Therefore, a reduction rather than inactivation of CS activity was preferable. Moreover, regulation of the *gltA* gene, which is responsible for 95% of CS activity in *C. glutamicum* [42], was widely applied to improve the biosynthesis of pyruvate-derived products [34, 43–45]. However, the impact of reducing CS activity on acetoin production is still unclear. Therefore, we used a weak promoter *P*_*1*_ with approximately 1% activity of the *P*_*tac*_ [46] to replace the native promoter of *gltA* gene in CGR6 and CGS1, yielding strains CGS7 and CGS8 respectively.

**Fig. 3:**
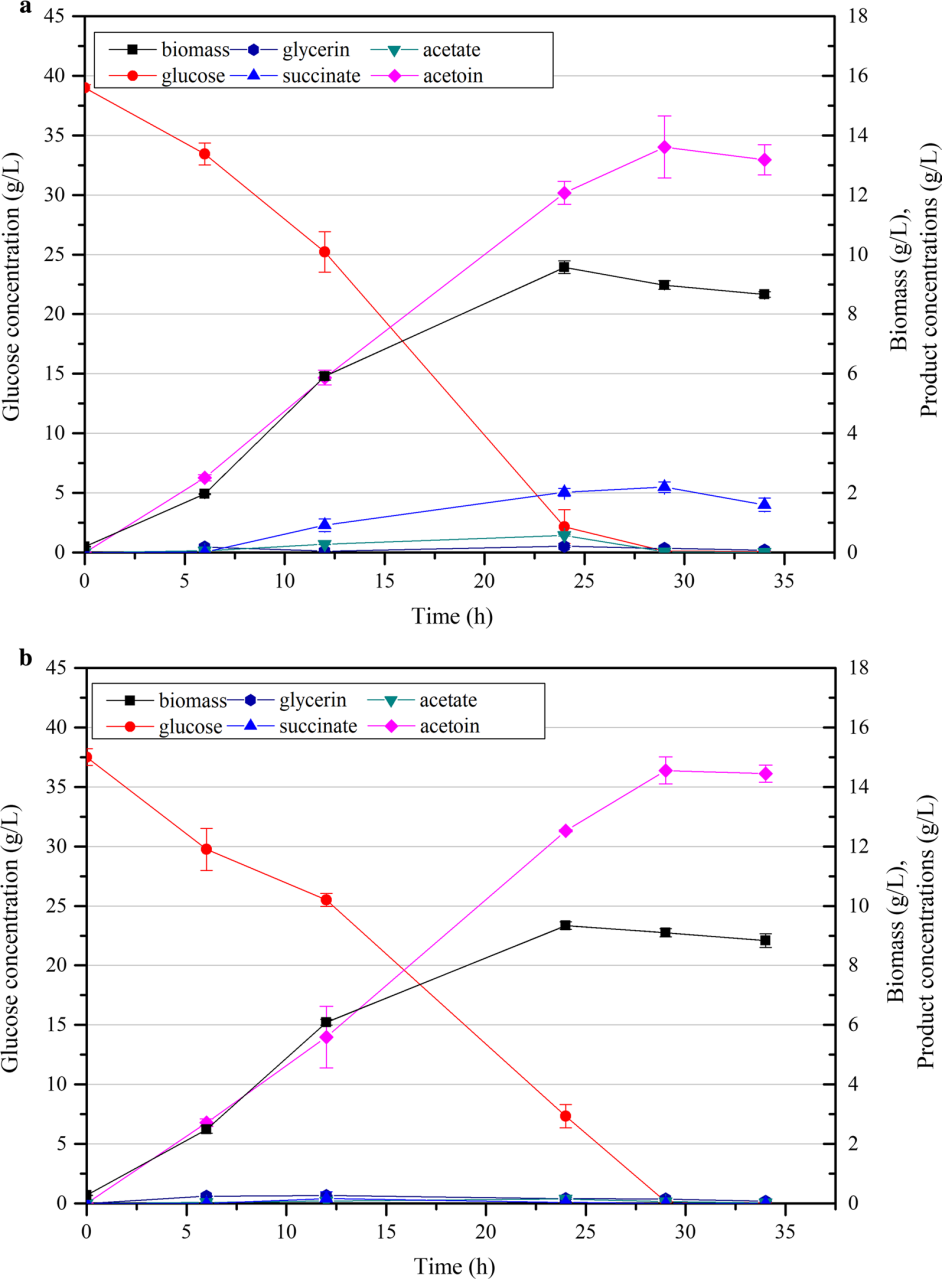
Time profiles of the biomass (g/L), glucose, organic acid and acetoin concentrations of strains CGS7 (**a**) and CGS8 (**b**) cultured with 38 g/L glucose. Error bars indicate the standard deviations from three independent cultures

The strains’ CS activity was measured to confirm its successful downregulation. As shown in Additional file 1: Fig. S3A, the CS activity of CGS7 at 12 h in the middle exponential phase was 75.8% lower than that of CGR6, and 60.0% lower at 22 h in the late exponential phase. As shown in Table 2, the succinate yield of CGS7 at 24 h was 20.0% lower than that of CGR6 (0.056 versus 0.070 g succinate/g glucose). Moreover, the cell growth and glucose consumption were also decreased (Fig. 3a), suggesting that the flux into the TCA cycle was indeed reduced. Unexpectedly, the acetate yield decreased by 46.7% (0.016 versus 0.030 g acetate/g glucose). We speculated that the decreased glucose consumption rate in CGS7 would reduce the fluxes to acetate synthesis caused by overflow metabolism, and the newly available carbon fluxes flowed to acetoin effectively, leading to an increase of the acetoin yield at 24 h in contrast to CGR6 (0.337 versus 0.302 g acetoin/g glucose). Then, the yield further increased to 0.350 g/g glucose with a titer of 13.61 g/L when glucose was depleted at 29 h (Fig. 3a). However, the results indicated that neither weakening *gltA* nor the deletion of *ppc* could effectively improve the intracellular pyruvate pool, but a combination of both eventually led to a 31.1% increase of intracellular pyruvate (Additional file 1: Fig. S3B). At the same time, the acetoin production was significantly enhanced to 14.56 g/L, with a yield of 0.389 g/g glucose when glucose was exhausted at 29 h, which was 18.6% higher than that of CGS1 (Fig. 3b). While deletion of *ppc* obviously inhibited glucose consumption of CGS8 compared to CGS7 (Table 2), however, the acetoin productivity was 0.52 g/L/h at 24 h, even higher than that of CGS7 (0.50 g/L/h). Moreover, the by-products acetate (0.15 g/L), glycerin (0.17 g/L) and succinate (0.03 g/L) remained at low concentrations. After considering acetoin production and by-product accumulation, CGS8 was selected for further engineering.

Furthermore, reduction of CS exhibited a fantastic synergistic effect on acetoin production with inactivation of PEP carboxylase, which is responsible for 90% of total oxaloacetate synthesis in *C. glutamicum* [47, 48]. As shown in Table 2, *ppc* knockout in CGS1 almost had no effect on acetoin yield (0.309 versus 0.302 g/g glucose), and *gltA* attenuation in CGS7 only led to a 11.6% increase in acetoin yield (0.337 versus 0.302 g/g glucose). However, the combination of the two gene modifications resulted in that the acetoin yield was significantly improved by 37.4% (0.415 versus 0.302 g/g glucose). The variations of intracellular pyruvate further confirmed the synergistic effect of CS and PEP carboxylase on acetoin production (Additional file 1: Fig. S3B), which would also be potential for improving other pyruvate-acetolactate-derived metabolites production.

### Effect of enhancing the acetoin synthesis pathway on acetoin production

To further pull the carbon flux from pyruvate toward product accumulation, two additional *alsSD* operon (under the control of the constitutive promoter *P*_*tuf*_) copies were inserted into the chromosome of CGS8 at the ∆*butA* and ∆*nagD* sites to generate the strain CGS9.

**Fig. 4:**
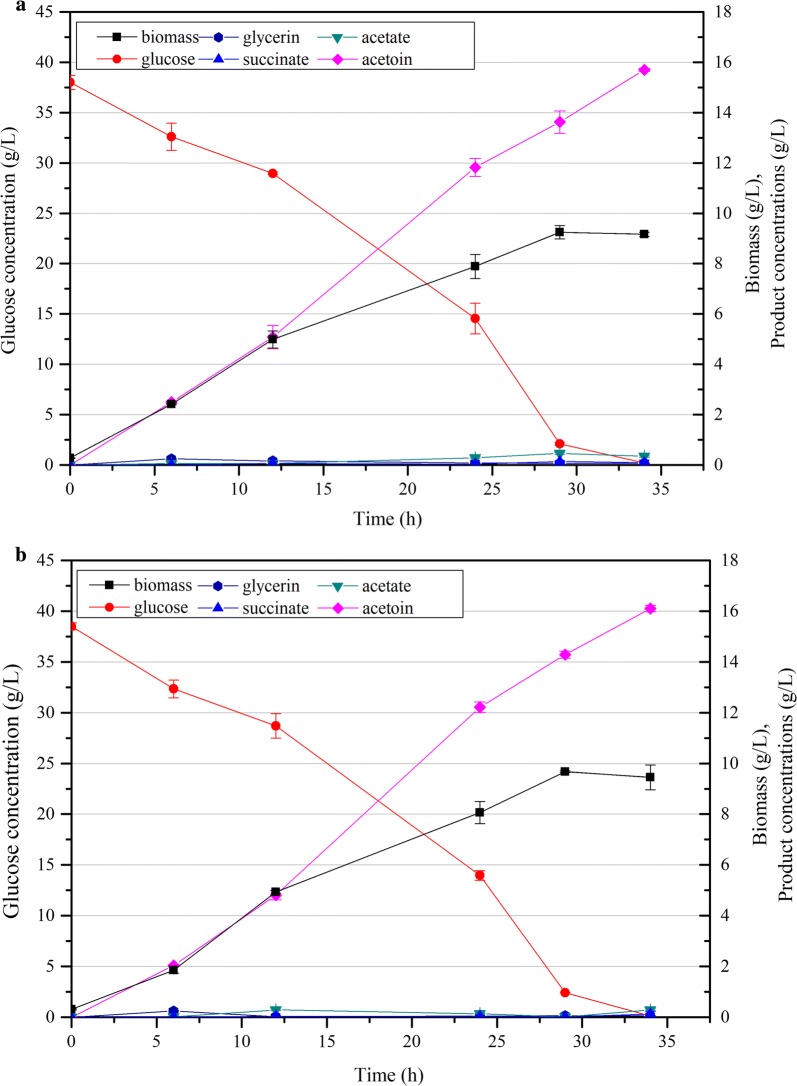
Time profiles of the biomass (g/L), glucose, organic acid and acetoin concentrations of strains CGS9 (**a**) and CGS11 (**b**) cultured with 38 g/L glucose. Error bars indicate the standard deviations from three independent cultures

The activities of ALS and ALDC were listed in Table 3. The ALS activity of CGS9 was 2.33-fold higher than that of CGR6 at 12 h in the middle log phase, and subsequently increased by 11.6% at 22 h in late log phase. However, the ALDC activity of CGS9 was increased by only 47.0% compared with that of CGR6 at 12 h. Then, it decreased by 15.1% at 22 h, but was still 37.3% higher than that of CGR6. With the increase of *alsSD* copies, the intracellular pyruvate concentration was slightly decreased by 8.2%. However, when glucose was exhausted at 34 h, 15.70 g/L acetoin was accumulated with a yield of 0.408 g/g glucose (Fig. 4a), which was merely 4.9% higher than that of CGS8. Therefore, the increased ALS and ALDC activity appeared to still be insufficient to increase acetoin production.

**Table 3:** Enzyme activities of different *C. glutamicum* strains

Strains	Middle log phase (12 h)		Late log phase (22 h)	
	ALS activity (U/mg)	ALDC activity (U/mg)	ALS activity (U/mg)	ALDC activity (U/mg)
CGR6	0.511 ± 0.087	0.608 ± 0.067	0.521 ± 0.060	0.553 ± 0.045
CGS9	1.192 ± 0.094	0.894 ± 0.044	1.330 ± 0.122	0.759 ± 0.054
CGS11	8.242 ± 0.362	2.864 ± 0.127	6.383 ± 0.778	1.074 ± 0.035

Error bars indicate the standard deviations from three independent cultures

To further pull pyruvate into acetoin synthesis pathway, the previously constructed *alsSD* over-expression plasmid pEC-XK99E-*alsSD*-Δ*lacIq* [9], was introduced into CGS9 to yield CGS11. Both the activities of ALS and ALDC were significantly improved compared with CGS9. Moreover, the intracellular pyruvate pool was further decreased by 6.7% compared with that of CGS9 (Additional file 1: Fig. S3B), indicating that more flux might have been re-directed toward acetoin synthesis. However, the acetoin production was still not obviously enhanced. When glucose was depleted at 34 h, the final acetoin titer was 16.10 g/L with a yield of 0.419 g/g glucose (Fig. 4b), which was only 7.7% higher than that of CGS8. Although the intracellular pyruvate pool of CGS11 was still 12.1% higher than that of CGR6, the carbon fluxes from pyruvate to competing pathways were successfully controlled as the titers of the main by-products, acetate (0.29 versus 1.12 g/L), glycerin (0.06 versus 0.23 g/L) and succinate (0.12 versus 2.63 g/L), were much lower than those of CGR6. No 2,3-butandiol or lactate was detected. Moreover, despite the decreases of cell growth and glucose consumption, the acetoin productivity was still comparable to that of strain CGS8. Therefore, strain CGS11 was a promising candidate for further scale-up fermentation.

Notably, both strain CGS9 and CGS11 showed much higher acetoin yields at 24 h (Table 2). The acetoin yield of strain CGS11 was even beyond the maximum theoretical yield of 0.489 g/g glucose and reached 0.498 g/g glucose at 24 h, which was mainly caused by the rich nutrients from the yeast extract in the CGXIIP medium. Moreover, all the detected by-products remained at low concentrations and biomass was relatively lower in CGS11 at 24 h (Table 2). Unexpectedly, the acetoin yield was then noticeably decreased to 0.419 g/g glucose at 34 h. The decreases of ALS and ALDC activities over time (Table 3) were initially suspected to be responsible for the decreased acetoin yield. However, the yields of by-products or biomass were not accordingly increased. Another possibility is that acetoin was re-used as an alternative carbon source when glucose was almost exhausted. Acetoin utilization is mainly catalyzed by the acetoin dehydrogenase (AoDH) complex [20, 49]. However, no candidate gene encoding a putative AoDH was identified in *C. glutamicum* through homologous sequence alignment (data not shown). Furthermore, acetoin degradation was more clearly observable when glucose was exhausted in fermentations of strains CGR6, CGS1 and CGS2 (Fig. 2a–c), but their biomass instead decreased during acetoin degradation. Thus, it did not appear that acetoin was consumed as a reserve carbon source, which would be expected to further support cell growth.

It is worth noting that two molecules of acetoin (C_4_H_8_O_2_) can be chemically converted to one molecule of 2,3,5,6-tetramethylpyrazine (TMP, C_8_H_12_N_2_, also called ligustrazine) in the presence of inorganic ammonium salts such as (NH_4_)_2_SO_4_ or diammonium phosphate [6, 30]. As shown in Additional file 1: Fig. S4, no TMP was accumulated in the fermentation broth of strain CGS11 at 24 h, but a titer of 1.27 g/L TMP was indeed detected at 34 h, which corresponded to a consumption of 1.64 g/L acetoin. Taking this portion of the generated acetoin into calculation, the yield could even reach a high level of 0.462 g/g glucose, which indicated that 94.4% of carbon fluxes had been directed toward acetoin synthesis.

### Fed-batch fermentation of CGS11 for efficient (3*R*)-acetoin production

**Fig. 5:**
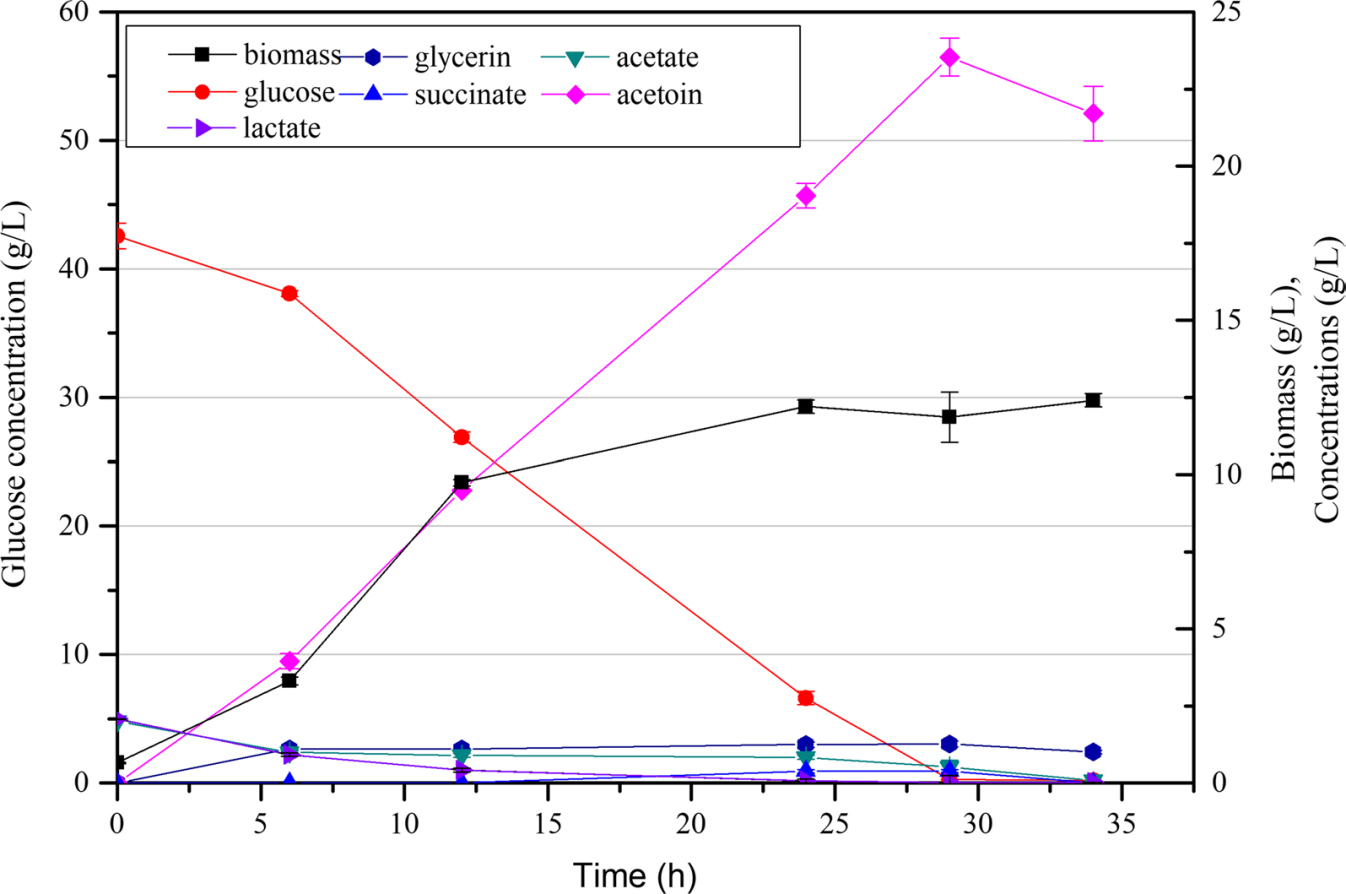
Time profiles of the biomass (g/L), glucose, organic acid and acetoin concentrations of strain CGS11 in LBRC medium in shake fermentation

After considering the promising shake-flask results, strain CGS11 was chosen for fed-batch fermentation in order to evaluate its potential for further industrial application. Before scale-up production, CGS11 was evaluated in the LBRC medium, which was optimized for acetoin production in *C. glutamicum* in our previous work [9]. As shown in Fig. 5, the acetoin production was significantly enhanced with a titer of 23.53 g/L (3*R*)-acetoin and a yield of 0.553 g/g glucose when glucose was exhausted at 29 h. The acetoin production in CGS11 was also much higher than that in our previous optimal strain CGR7 (17.10 g/L (3*R*)-acetoin with a yield of 0.428 g/g glucose from LBRC medium) [9]. The rich nutrients such as lactate (initial 2.07 g/L) and amino acids from corn steep liquor (CSL) in LBRC medium should be the major reason for the improved acetoin production. And the remarkable effect on acetoin production with CSL addition was consistent with previous report in *B. subtilis* [50, 51]. Moreover, when using organic nitrogen source to replace inorganic nitrogen source (NH_4_)_2_SO_4_, no TMP was detected (Additional file 1: Fig. S4), which might be another reason for the improved acetoin production. Given that CSL is cheap and abundant, the cost caused by addition of CSL could be easily made up by the significant enhancement of acetoin production. The LBRC medium was therefore adopted for further fed-batch fermentation.

**Fig. 6:**
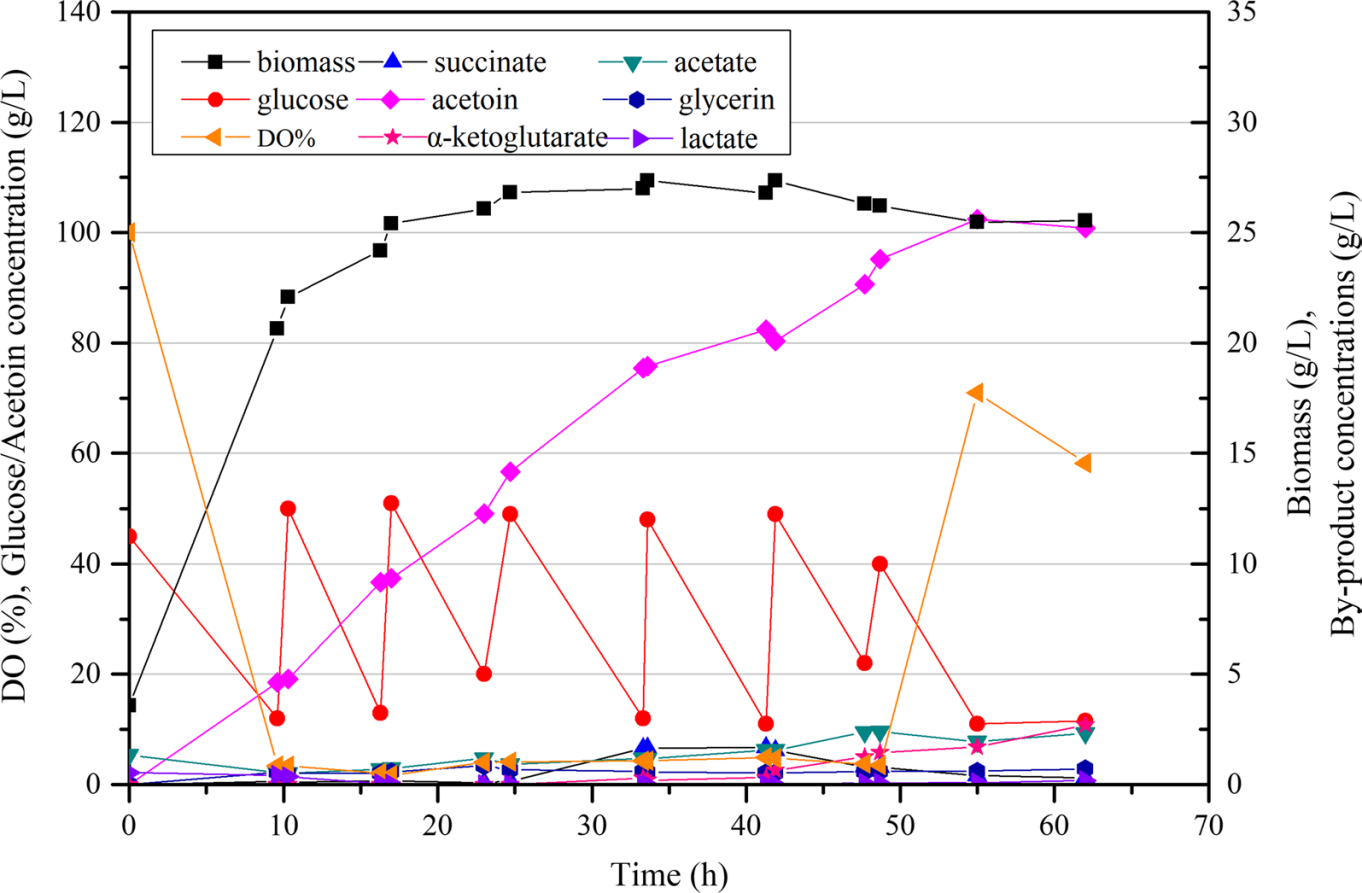
(3*R*)-acetoin production from glucose using CG11 in fed-batch fermentation. The strain was cultured in LBRC medium with initial 50 g/L glucose at 30 °C and 600 rpm in a 5-L fermenter under aeration of 1 vvm. A stock solution comprising 1000 g/L glucose was added when the glucose concentration dropped below 15 g/L to keep the glucose concentration between 10 and 50 g/L

During the fed-batch fermentation process, the cell growth, residual glucose, product concentrations and relative dissolved oxygen (DO) were determined. As shown in Fig. 6, a titer of 102.45 g/L acetoin was obtained at 55 h, corresponding to an average productivity of 1.86 g/L/h. The final fermentation volume was 2.45 L and a total of 625.85 g glucose was added in 7 batches, while the remaining glucose amount at 55 h was 11 g/L. Therefore, the acetoin yield was 0.419 g/g glucose, reaching 85.7% of the theoretical yield. The optical purity of the produced (3*R*)-acetoin surpassed 95% (Additional file 1: Fig. S5), which was consistent with our previous results [9]. No TMP was detected during the entire fed-batch fermentation (Additional file 1: Fig. S4). The final concentrations of acetate, succinate, glycerin and lactate were 1.94, 0.60, 0.41 and 0.33 g/L, respectively. However, α-ketoglutarate, which was undetectable during batch fermentation in shake flask, started to accumulate after 33 h and reached a final concentration of 1.71 g/L at 55 h. Nevertheless, all the by-products remained at acceptably low concentrations.

We noticed that the glucose consumption and acetoin production were prone to stop when acetoin titer surpassed 100 g/L (from 55 to 62 h, as shown in Fig. 6). With an initial acetoin concentration of 80 g/L in shake fermentation, the specific growth rate of *B. amyloliquefaciens* was decreased by 99% [52], while wild-type *B. subtilis* 168 was even unable to grow on plates containing 50 g/L acetoin [53]. Therefore, the toxicity of the very high acetoin concentrations will no doubt prevent glucose assimilation and acetoin production of CGS11, and even activate its underlying acetoin catabolism. Since acetoin metabolism is complex and still not clearly elucidated, several successful strategies, such as physical/chemical mutagenesis [54], adaptive evolution [52, 53] or omics focusing on global transcriptional/metabolism level responses to acetoin stress [55], could be adopted to deeply understand and improve the acetoin tolerance of *C. glutamicum* in future studies.

To our best knowledge, the value of 102.45 g/L is the highest titer of highly enantiomerically enriched (3*R*)-acetoin reported to date (Table 4), as well as the best result obtained for acetoin production via microbial fermentation. Moreover, the yield and productivity were also good enough to merit further industrial application. Furthermore, the strains engineered in this work could also be used as platform strains to be further developed as microbial cell factories for production of (3*S*)-acetoin and chiral 2,3-butanediol.

**Table 4:** Comparison of the production of acetoin via microbial fermentation process in the literature and in this study

Strains/enzymes	Substrate	Titer (g/L)	Enantiomer	Yield (g/g)	Productivity (g/L/h)	Refs.
Production of optically pure acetoin						
*C. glutamicum* CGS11	Glucose	102.45	(3*R*)-	0.419	1.86	This study
*C. glutamicum* CGR7	Glucose	96.20	(3*R*)-	0.360	1.30	[9]
*Bacillus subtilis* CGMCC 13141	Glucose	83.70	(3*R*)-	0.448	1.02	[25]
*Klebsiella pneumoniae*	Glucose	62.30	(3*R*)-	0.140	1.09	[20]
*E. coli* (pAC-NOX)	Glucose	60.30	(3*R*)-	0.422	1.55	[58]
*Bacillus subtilis* DL01	Sugarcane molasses	58.20	(3*R*)-	0.291	0.77	[59]
*Lactococcus lactis* CS4701m	Glucose	5.80	(3*S*)-	0.347	0.19	[23]
Production of racemic acetoin (or optical purity not stated)						
*S. cerevisiae* JHY617-SDN	Glucose	100.10	Ng	0.440	1.82	[16]
*Gluconobacter oxydans* DSM 2003	2,3-Butanediol	89.20	Ng	0.891	1.24	[60]
*B. subtilis* HS019	Glucose	82.50	Ng	0.460	1.33	[53]
*Bacillus licheniformis* WX-02*ΔbudCΔacoR*	Glucose	78.80	Ng	0.310	0.58	[7]
*S. marcescens* H32-*nox*	Sucrose	75.20	Ng	0.360	1.88	[61]
*Enterobacter aerogenes* EJW-03	Glucose	71.70	Ng	0.320	2.87	[62]
*B. amyloliquefaciens* E-11	Glucose	71.50	Ng	0.413	1.63	[52]
*B. licheniformis* MW3 *(*△*budC*△*gdh)*	Glucose	64.20	Ng	0.412	2.38	[63]
*B. subtilis* ZB02	Glucose, xylose, and arabinose	62.20	Ng	0.288	0.86	[64]
*S. marcescens* H32	Sucrose	60.50	Ng	Ng	1.44	[65]
*B. subtilis* F126-2	Glycerol	60.48	Ng	Ng	1.26	[66]
*Lactococcus lactis* CML B4	Glucose	59.00	Ng	0.360	2.11	[67]
*Paenibacillus polymyxa* CJX518	Glucose	57.20	Ng	0.400	0.30	[68]
*B. subtilis* TH-49	Glucose	56.90	Ng	Ng	0.89	[69]
*B. subtilis* BMN	Glucose	56.70	Ng	0.378	0.68	[70]
*P. polymyxa* CS107	Glucose	55.30	Ng	0.370	1.32	[71]
*B. subtilis* JNA-UD-6	Glucose	53.90	Ng	0.359	0.37	[72]
*Enterobacter cloacae* SDM 45	Glucose	55.20	Ng	0.373	2.69	[73]
	Lignocellulosic hydrolysate	45.60	Ng	NG	1.52	
*B. amyloliquefaciens* FMME044	Glucose	51.20	Ng	0.430	1.42	[74]
*Bacillus sp.* H15-1.	Degermed maize flour hydrolysate	50.80	Ng	0.285	NG	[75]
*C. glutamicum* CGT2	Glucose	40.51	Ng	0.240	0.51	[76]

Ng indicates that the related information is Not Given

## Conclusions

In summary, this is the first report on reducing CS activity to improve acetoin production. Moreover, combination of attenuation of CS and inactivation of PEP showed a significant synergistic effect on acetoin production. The highly engineered *C. glutamicum* strain CGS11 produced a titer of 102.45 g/L acetoin with a yield of 0.419 g/g glucose at a rate of 1.86 g/L/h in a 5 L fermenter, which is the highest titer of highly enantiomerically enriched (3*R*)-acetoin together with a competitive product yield and productivity, which opens the possibility of finally realizing the promises of industrial (3*R*)-acetoin production via green chemical process in the near future.

## Supplementary information

**Additional file 1: Table S1** Primers used in this study. **Fig. S1.** Growth curves of strains CGS3, CGS4, CGS5, CGS6 and CGR6 in CGXIIY medium (per liter) containing 1 g yeast extract, 5 g (NH_4_)_2_SO_4_, 5 g urea, 1 g KH_2_PO_4_, 1 g K_2_HPO_4_, 0.25 g MgSO_4_·7H_2_O, 0.01 g CaCl_2_, 0.01 g FeSO_4_·7H_2_O, 0.1 mg MnSO_4_·H_2_O, 1 mg ZnSO_4_·7H_2_O, 0.2 mg CuSO_4_·5H_2_O, 0.02 mg NiCl·6H_2_O and 0.4 mg biotin and 40 g glucose, pH 7.0. **Fig. S2.** Time profiles of the biomass (g/L), glucose, organic acid and acetoin concentrations of strain CGS5 in CGXIIP medium. **Fig. S3.** (A) The activity of citrate synthase in CGR6 and CGS7. (B) The intracellular pyruvate concentrations of different strains at 12 h. Error bars indicate the standard deviations from three independent cultures. **Fig. S4.** Measurement of 2,3,5,6-tetramethylpyrazine concentrations by GC-FID. A: The 2,3,5,6-tetramethylpyrazine standard had a retention time of 10.598 min; B: Batch fermentation products of CGS11 in CGXIIP medium at 24 h; C: Batch fermentation products of CGS11 in CGXIIP medium at 34 h; D: Batch fermentation products of CGS11 in LBRC medium at 29 h; E: Fed-batch fermentation products of CGS11 in LBRC medium at 55 h. **Fig. S5.** Identification of acetoin enantiomers by GC-FID. A: The optically pure standards of (3*R*)- and (3*S*)- had retention times of 9.215 and 9.598 min, respectively; B: Fermentation products of CGS11 in CGXIIP medium; C: Fed-batch fermentation products of CGS11 in LBRC medium at 55 h.
