# Supplementary material for: Engineering central pathways for industrial-level (3R)-acetoin biosynthesis in Corynebacterium glutamicum
Source: Microb Cell Fact. 2020 May 12;19:102. doi: 10.1186/s12934-020-01363-8 (PMC7216327; doi:10.1186/s12934-020-01363-8)
Supplement: Supplementary file 1 — Additional file 1: Table S1 Primers used in this study. Fig. S1. Growth curves of strains CGS3, CGS4, CGS5, CGS6 and CGR6 in CGXIIY medium (per liter) containing 1 g yeast extract, 5 g (NH4)2SO4, 5 g urea, 1 g KH2PO4, 1 g K2HPO4, 0.25 g MgSO4·7H2O, 0.01 g CaCl2, 0.01 g FeSO4·7H2O, 0.1 mg MnSO4·H2O, 1 mg ZnSO4·7H2O, 0.2 mg CuSO4·5H2O, 0.02 mg NiCl·6H2O and 0.4 mg biotin and 40 g glucose, pH 7.0. Fig. S2. Time profiles of the biomass (g/L), glucose, organic acid and acetoin concentrations of strain CGS5 in CGXIIP medium. Fig. S3. (A) The activity of citrate synthase in CGR6 and CGS7. (B) The intracellular pyruvate concentrations of different strains at 12 h. Error bars indicate the standard deviations from three independent cultures. Fig. S4. Measurement of 2,3,5,6-tetramethylpyrazine concentrations by GC-FID. A: The 2,3,5,6-tetramethylpyrazine standard had a retention time of 10.598 min; B: Batch fermentation products of CGS11 in CGXIIP medium at 24 h; C: Batch fermentation products of CGS11 in CGXIIP medium at 34 h; D: Batch fermentation products of CGS11 in LBRC medium at 29 h; E: Fed-batch fermentation products of CGS11 in LBRC medium at 55 h. Fig. S5. Identification of acetoin enantiomers by GC-FID. A: The optically pure standards of (3R)- and (3S)- had retention times of 9.215 and 9.598 min, respectively; B: Fermentation products of CGS11 in CGXIIP medium; C: Fed-batch fermentation products of CGS11 in LBRC medium at 55 h. [file 12934_2020_1363_MOESM1_ESM.doc]

**Engineering** **central pathways for industrial-level (3*R*)-acetoin biosynthesis in *Corynebacterium glutamicum***

Lingxue Lu^1, †^, Yufeng Mao^2, †^, Mengyun Kou^1^, Zhenzhen Cui^1^, Biao Jin^1^, Zhishuai Chang^1^, Zhiwen Wang^1^, Hongwu Ma^2^, Tao Chen^1, *^

^1^ Frontier Science Center for Synthetic Biology and Key Laboratory of Systems Bioengineering of Ministry of Education, SynBio Research Platform, Collaborative Innovation Center of Chemical Science and Engineering, School of Chemical Engineering and Technology, Tianjin University, Tianjin 300072, China.

^2^ Key Laboratory of Systems Microbial Biotechnology, Tianjin Institute of Industrial Biotechnology, Chinese Academy of Sciences, Tianjin 300308, China.

* Corresponding author: Tao Chen

E-mail: chentao@tju.edu.cn

Address: Department of Biochemical Engineering, School of Chemical Engineering and Technology, Tianjin University, Tianjin 300072, People’s Republic of China.

† These authors contributed equally to this work.

**Supplementary Tables**

**Table S1 Primers used in this study**

| Primers | Sequence | Purpose |
| --- | --- | --- |
| pyc-1 | TCATAGGATCCTGAGCCCCATCCGTTTGAAGACTGT | To construct pD-*sacB*-*pyc* |
| pyc-2 | CAGCCACCTGGAGGGTTACCAAGCTCAACAAGAGACCGCCAAGGGTGATAGC | To construct pD-*sacB*-*pyc* |
| pyc-3 | GCTATCACCCTTGGCGGTCTCTTGTTGAGCTTGGTAACCCTCCAGGTGGCTG | To construct pD-*sacB*-*pyc* |
| pyc-4 | CGGATGTCGACATCAAGTCGCCACCTTCCACCTTCG | To construct pD-*sacB*-*pyc* |
| ICD^A94D^-1 | CGTAAGGATCCGAACAGATCACAGAATCCAACCCACG | To construct PD-*sacB*-*icd*-mut-1 |
| ICD^A94D^-2 | GTCCTGCAGTTCCTTAATATCAGCCTTGAGCTGTGGAAC | To construct PD-*sacB*-*icd*-mut-1 |
| ICD^A94D^-3 | GTTCCACAGCTCAAGGCTGATATTAAGGAACTGCAGGAC | To construct PD-*sacB*-*icd*-mut-1 |
| ICD^A94D^-4 | GCATTGTCGACGAAGATGATTGGGTCGGAGA | To construct PD-*sacB*-*icd*-mut-1 |
| ICD^G407S/R453C^-1 | GTTAAGAATTCCCCGGGGACGCTGCTGATGAAGTTC | To construct PD-*sacB*-*icd*-mut-2 and PD-*sacB*-*icd*-mut-3 |
| ICD^G407S^-2 | CTTCAGCCTTCTGAGCCATCAGACTAACGTTAGGGACGGTACCCATGGTG | To construct PD-*sacB*-*icd*-mut-2 |
| ICD^G407S^-3 | CACCATGGGTACCGTCCCTAACGTTAGTCTGATGGCTCAGAAGGCTGAAG | To construct PD-*sacB*-*icd*-mut-2 and PD-*sacB*-*icd*-mut-3 |
| ICD^G407S/R453C^-4 | CATTGGTCGACCTGTGTTCAGTGCTTCTGCGACTGGT | To construct PD-*sacB*-*icd*-mut-2 |
| ICD^R453C^-2 | CGACGTTGAGGCAAATGACATCTGGTGTGCATGCCAGGTCAAGG | To construct PD-*sacB*-*icd*-mut-3 |
| ICD^R453C^-3 | CCTTGACCTGGCATGCACACCAGATGTCATTTGCCTCAACGTCG | To construct PD-*sacB*-*icd*-mut-3 |
| BalsSD-1 | TTCGACTCGAGTGGCCGTTACCCTGCGAATGTC | To construct PD-*sacB*-*butA*-*alsSD* |
| BalsSD-2 | GGTTCGTCGACTTATTCAGGGCTTCCTTCAGTTGTT | To construct PD-*sacB*-*butA*-*alsSD* |
| NalsSD-1 | ATCGACTCGAGTGGCCGTTACCCTGCGAATGTC | To construct PD-*sacB*-*nagD*-*alsSD* |
| NalsSD-1 | GGTTCACTAGTTTATTCAGGGCTTCCTTCAGTTGTT | To construct PD-*sacB*-*nagD*-*alsSD* |

**Supplementary Figures**





**Figure S1** Growth curves of strains CGS3, CGS4, CGS5, CGS6 and CGR6 in CGXIIY medium (per liter) containing 1 g yeast extract, 5 g (NH_4_)_2_SO_4_, 5 g urea, 1 g KH_2_PO_4_, 1 g K_2_HPO_4_, 0.25 g MgSO_4_·7H_2_O, 0.01 g CaCl_2_, 0.01 g FeSO_4_·7H_2_O, 0.1 mg MnSO_4_·H_2_O, 1 mg ZnSO_4_·7H_2_O, 0.2 mg CuSO_4_·5H_2_O, 0.02 mg NiCl·6H_2_O and 0.4 mg biotin and 40 g glucose, pH 7.0.





**Figure S2** Time profiles of the biomass (g/L), glucose, organic acid and acetoin concentrations of strain CGS5 in CGXIIP medium.








**Figure S3** (A) The activity of citrate synthase in CGR6 and CGS7. (B) The intracellular pyruvate concentrations of different strains at 12 h. Error bars indicate the standard deviations from three independent cultures.


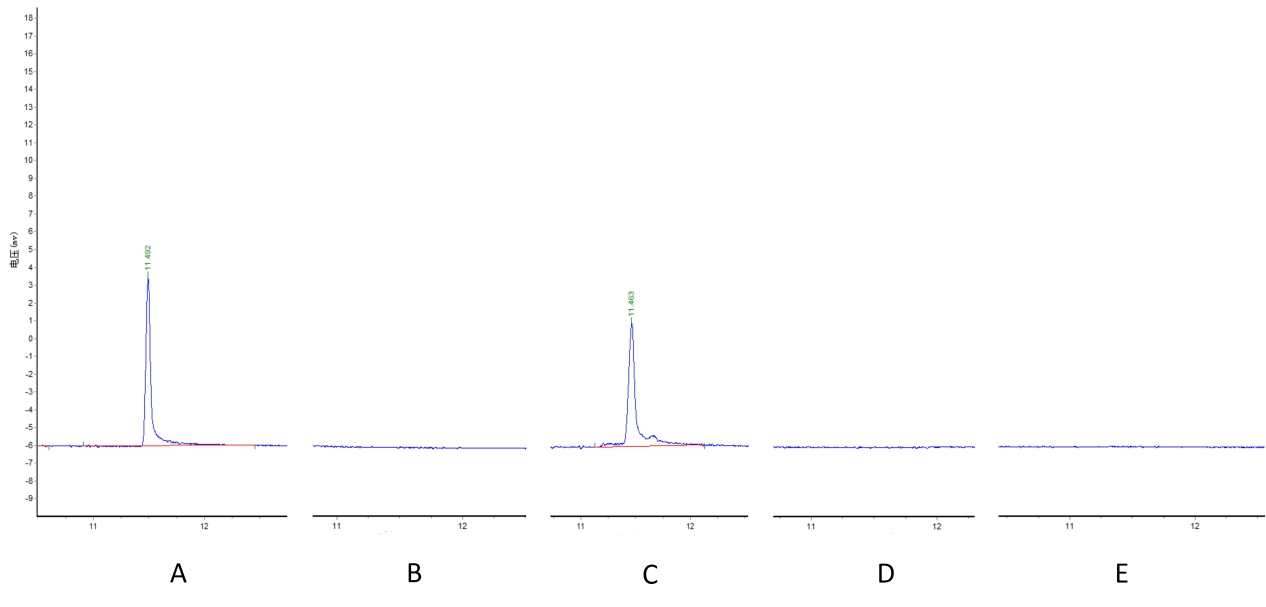


**Figure S4** Measurement of 2,3,5,6-tetramethylpyrazine concentrations by GC-FID. A: The 2,3,5,6-tetramethylpyrazine standard had a retention time of 10.598 min; B: Batch fermentation products of CGS11 in CGXIIP medium at 24 h; C: Batch fermentation products of CGS11 in CGXIIP medium at 34 h; D: Batch fermentation products of CGS11 in LBRC medium at 29 h; E: Fed-batch fermentation products of CGS11 in LBRC medium at 55 h.


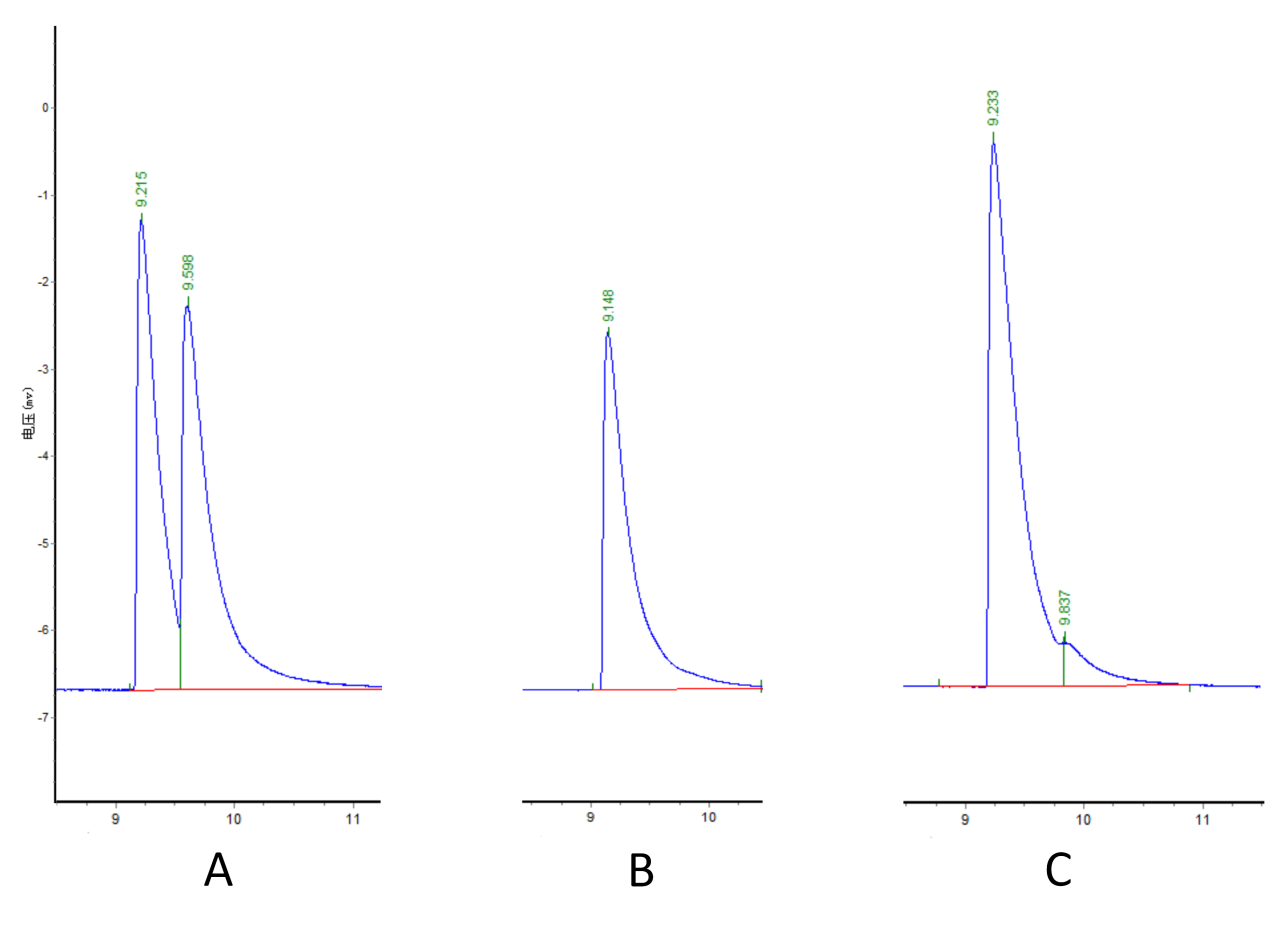


**Figure S5** Identification of acetoin enantiomers by GC-FID. A: The optically pure standards of (3*R*)- and (3*S*)- had retention times of 9.215 and 9.598 min, respectively; B: Fermentation products of CGS11 in CGXIIP medium; C: Fed-batch fermentation products of CGS11 in LBRC medium at 55 h.
